# Lyophilized allografts without pre-treatment with glutaraldehyde are more suitable than cryopreserved allografts for pulmonary artery reconstruction

**DOI:** 10.1590/1414-431X20155001

**Published:** 2015-12-04

**Authors:** J.R. Olmos-Zúãiga, R. Jasso-Victoria, N.E. Díaz-Martínez, M.O. Gaxiola-Gaxiola, A. Sotres-Vega, Y. Heras-Romero, M. Baltazares-Lipp, M.E. Baltazares-Lipp, P. Santillán-Doherty, C. Hernández-Jiménez

**Affiliations:** 1 Department of Experimental Surgery, National Institute of Respiratory Diseases "Ismael Cosío Villegas", Mexico City, Mexico; 2 Medical and Pharmaceutical Biotechnology, Center for Research and Assistance in Technology and Design of the State of Jalisco, Guadalajara, Jalisco, Mexico; 3 Laboratory of Morphology, National Institute of Respiratory Diseases "Ismael Cosío Villegas", Mexico City, Mexico; 4 Hemodynamics and Echocardiography Service, National Institute of Respiratory Diseases "Ismael Cosío Villegas", Mexico City, Mexico; 5 Medical Administration, National Institute of Respiratory Diseases "Ismael Cosío Villegas", Mexico City, Mexico

**Keywords:** Pulmonary artery graft, Cryopreservation, Lyophilization, Blood vessel bioprosthesis, Prosthesis design, Vascular procedures

## Abstract

Various methods are available for preservation of vascular grafts for pulmonary artery (PA) replacement. Lyophilization and cryopreservation reduce antigenicity and prevent thrombosis and calcification in vascular grafts, so both methods can be used to obtain vascular bioprostheses. We evaluated the hemodynamic, gasometric, imaging, and macroscopic and microscopic findings produced by PA reconstruction with lyophilized (LyoPA) grafts and cryopreserved (CryoPA) grafts in dogs. Eighteen healthy crossbred adult dogs of both sexes weighing between 18 and 20 kg were used and divided into three groups of six: group I, PA section and reanastomosis; group II, PA resection and reconstruction with LyoPA allograft; group III, PA resection and reconstruction with CryoPA allograft. Dogs were evaluated 4 weeks after surgery, and the status of the graft and vascular anastomosis were examined macroscopically and microscopically. No clinical, radiologic, or blood-gas abnormalities were observed during the study. The mean pulmonary artery pressure (MPAP) in group III increased significantly at the end of the study compared with baseline (P=0.02) and final [P=0.007, two-way repeat-measures analysis of variance (RM ANOVA)] values. Pulmonary vascular resistance of groups II and III increased immediately after reperfusion and also at the end of the study compared to baseline. The increase shown by group III *vs* group I was significant only if compared with after surgery and study end (P=0.016 and P=0.005, respectively, two-way RM ANOVA). Microscopically, permeability was reduced by ≤75% in group III. In conclusion, substitution of PAs with LyoPA grafts is technically feasible and clinically promising.

## Introduction

Various diseases (e.g., congenital malformations, tumors, strictures, and injuries) affect the pulmonary artery (PA), necessitating treatment by resection and reconstruction with vascular grafts to maintain cardiopulmonary function (1). Some synthetic materials (e.g., polyethylene terephthalate [Dacron¯] and polytetrafluoroethylene) and biologic materials (e.g., autologous or bovine pericardium) have been used as grafts for PA reconstruction, with differing results. Tissue engineering has been used with these materials and showed several disadvantages: growth failure; loss of mechanical strength over time; little capacity for remodeling and regeneration; calcification formation; increased risk of infection; aneurysm formation (2); high cost; limited availability in "developing" countries. In this regard, vascular prostheses have been obtained with cell cultures on synthetic or biologic matrices, but without achieving the desired success (3-5).

Tissue-preservation methods have also been used, such as lyophilization and cryopreservation, which reduce antigenicity and prevent thrombosis and calcification of the graft. Lyophilization is a physical process by which cells or tissues are dehydrated, providing long-term preservation and maintenance of graft function. It has been found that use of lyophilization, without pretreatment, does not alter the mechanical characteristics of biomaterials and improves their immunogenic properties, reduces calcification, and allows graft storage for future use. Conversely, cryopreservation of vascular tissues has been shown to lead to partial loss of the function and tissue of the endothelium, as well as the contractility of smooth muscle. Moreover, cryopreservation seems to be associated with increased influx of Ca^2+^ through dihydropyridine-sensitive Ca^2+^ channels (6-9).

Cryopreserved allografts and lyophilized bovine pericardium grafts treated with glutaraldehyde have been used for the replacement of heart valves. However, grafts pretreated with glutaraldehyde can develop calcifications. Taken together, it could be assumed that lyophilized PAs, without pretreatment, would maintain their mechanical properties, enhance their immunogenic properties, prevent graft calcification, and would not require anticoagulation therapy.

We wished to evaluate the hemodynamic, gasometric, imaging, macroscopic, and microscopic changes produced by PA reconstruction with lyophilized and cryopreserved PA grafts in dogs, and any possible outcome differences for both methods. This is the first study to compare the effects of these preservation methods under identical conditions.

## Material and Methods

### Experimental animals

This experimental study was designed in the Department of Experimental Surgery, National Institute of Respiratory Diseases "Ismael Cosío Villegas" (INER), Mexico. Twenty-four healthy crossbred adult dogs (male and female; 18-20 kg) were used. Dogs were chosen as experimental animals because their physiology and cardiovascular anatomy are similar to those of humans. In addition, their pulmonary hilum is readily accessible and long, whereas further development of the digestive system in pigs and sheep causes significant differences compared with human hemodynamics. Before the experiment, dogs were confined to individual cages (1.0 m×3.5 m×2.7 m) (10) under identical environmental conditions. Animals had water and food *ad libitum*. The study protocol was approved by the Bioethics Committee of INER (protocol number, B12-06). All animals were treated in strict accordance with the Technical Specifications for the Care and Use of Laboratory Animals of the Mexican Official Standard NOM-062-ZOO-1999 (10) and the Guide for the Care and Use of Laboratory Animals of the United States of America (11). Sample size was reduced in agreement with the principles of experimental methods proposed by Kilkenny et al. (12).

### Study groups

Eighteen animals were divided into three study groups of six dogs each and subjected to left pulmonary artery (LPA) reconstruction: group I: LPA section and end-to-end anastomosis; group II: LPA resection and reconstruction with a lyophilized pulmonary artery (LyoPA) graft; group III: LPA resection and reconstruction with a cryopreserved pulmonary artery (CryoPA) graft. The remaining six dogs were graft donors.

### Anesthesia and surgical procedure

In all animals, anesthesia was induced by intravenous injection of 0.1 mg/kg body weight xylazine hydrochloride (Rompun¯, Bayer, Germany) and 6 mg/kg propofol (Diprivan¯, AstraZeneca, Mexico). Endotracheal intubation was undertaken (Rush Kamunting, Malaysia) and general anesthesia was maintained with 1.5% isoflurane (Abbott, Mexico).

### Surgical method in donor dogs

The anesthetized dog was placed in the dorsal decubitus position. A median sternotomy was carried out, with dissection of the aorta, cava veins, and trachea. After systemic administration of heparin (20 IU/kg *iv*), animals were euthanized with an overdose (80 mg/kg *iv*) of sodium pentobarbital (Anestesal¯, Pfizer, Mexico), and the cardiopulmonary block removed. Both PAs were obtained immediately from their origin in the heart and at the entrance of the upper lobe. Then, they were washed with 100 mL of 0.9% saline solution supplemented with 5000 IU of heparin (Inhepar¯, Pisa, Mexico), 1,000,000 IU of procaine penicillin (Pisa), and 1 g of streptomycin sulfate (Sulfastrep¯, Pisa) per 500 mL of solution.

### LyoPA preparation

Before lyophilization, the PA trunk was cut for use as a histologic control. A silicone cannula was introduced in each branch of the artery (to maintain tubular structure during lyophilization). Then, they were placed into a flask and frozen at -70°C for 24 h. Subsequently, specimens were lyophilized in a vacuum of 10 mbar at -55°C for 4 h in a FreeZone 6 Liter Benchtop Freeze Dry System (Labconco, USA). Next, the cannulas were removed, and each PA was packaged and sterilized with a Sterrad¯ Hydrogen Peroxide Gas Plasma Sterilization System (ASP, USA). Then, each PA underwent low-temperature sterilization with hydrogen peroxide gas plasma (Johnson & Johnson Medical, USA) and stored at room temperature until use.

### CryoPA preparation

After procurement and washing of grafts, clots were removed from all PAs. The PA trunk was cut but not subjected to cryopreservation because it was used as a histologic control. Subsequently, each PA was placed in a cryotube with CS-C medium specific for endothelial cells (C1431, Sigma-Aldrich, USA) supplemented with endothelial cell growth factor (E9640, Sigma-Aldrich), 10% dimethyl sulfoxide (DMSO; D2650, Sigma-Aldrich), 20% fetal bovine serum (FBS; 16000-044, Gibco, USA), and antibiotic-antimycotic solution (A5955, Sigma-Aldrich). Cryotubes were placed in a polystyrene box to freeze gradually at -1°C/min until they reached -70°C in the freezer, where they remained for 48 h before placement in liquid nitrogen (-196°C vapor phase) for 15 days until use.

### Surgical procedure in recipient dogs

Before graft placement, a 5-F thermodilution catheter (Swan-Ganz Standard Thermodilution Balloon Catheter, Edwards Lifesciences, Canada) was introduced into the right jugular vein in all dogs and positioned in the PA trunk for hemodynamic measurement with a vital signs monitor (Datascope Passport, USA) and for measurement of cardiac output with a hemodynamic profile computer (SP1445, Spectramed, USA). A catheter was also placed in the right carotid artery to record systemic parameters. Samples for blood gas analyses were drawn through these catheters, which were then processed with a gas analyzer (AVL Compact 2, Graz, Australia).

For graft placement, a left thoracotomy was carried out at the fifth intercostal space by dissection of the pulmonary hilum. In group I, the LPA was sectioned and a reanastomosis done using a continuous 4-0 polypropylene suture (Prolene¯, Ethicon, USA). In groups II and III, a 1.5-cm segment of the LPA was excised and replaced with the LyoPA graft and CryoPA graft, respectively, using the same pattern and suture material. All animals received the antibiotic enrofloxacin (5 mg/kg, *im*; Baytril¯, Bayer, Germany) and the analgesic flunixin meglumine (0.1 mg/kg, *im*; Napzin¯, Pisa) for 5 days after surgery. Dogs were allowed to recover in their cages and were maintained for 4 weeks postoperatively. No animal received immunosuppressive or anticoagulation therapy over the course of the study.

Before initiation of the surgical procedure, the LyoPA graft was rehydrated for 30 min in saline solution at 37°C. The CryoPA graft was thawed for 30 min by placing the cryovial in a water-bath at 37°C. Then, the CryoPA graft was washed with saline at 37°C. The same surgeon carried out all surgical procedures.

### Surgical management

Surgical management of grafts was evaluated according to their consistency, ease of passage of the suture, ability to regain form during reperfusion, and hemostatic ability in suture sites.

### Microbiological cultures

Upon conclusion of preservation and before placement of grafts in PAs, all grafts underwent microbiologic analyses for isolation of bacteria and fungi.

### Clinical evaluation

Dogs were evaluated clinically every day during the first week after surgery and every other day for the remaining 3 weeks of the study. We focused mainly on the severity of dyspnea evaluated according to the Medical Research Council Modified Scale: 0, without shortness of breath after running; 1, shortness of breath after running; 2, difficulty in breathing at rest.

### Hemodynamics and blood gas analyses

Hemodynamics and blood gas analyses were carried out before surgery, immediately after placement of the graft in the LPA, and at study end. Evaluated parameters were: heart rate, cardiac output, mean pulmonary artery pressure (MPAP), pulmonary capillary pressure, systemic mean arterial pressure, central venous pressure, pulmonary vascular resistance (PVR), systemic vascular resistance, shunt, partial pressure of oxygen, partial pressure of carbon dioxide, and arterial and venous pH.

### Imaging studies

Ventrodorsal radiographs were taken preoperatively and every week to observe the lungs and thoracic cavity. At study end, graft patency was assessed by ventrodorsal angiography and transthoracic echocardiography (Vivid FiVe™, General Electric, USA).

### Macroscopic and microscopic studies

Upon rehydration or thawing of the graft, its overall state was assessed by identification of ruptures or fractures.

Four weeks after surgery, dogs were euthanized with an overdose of sodium pentobarbital (Anestesal¯, Pfizer). Status of the anastomosis in the graft, its integration, healing, presence of thrombi or stenoses, aneurysm dehiscence, and infection or necrosis were evaluated. Then, the grafts were excised.

For histologic evaluation, specimens were fixed in 10% formalin, embedded in paraffin, and stained with hematoxylin-eosin and Masson's trichrome. The structural integrity, inflammation, and atrophy of the PA were evaluated under light microscopy. In addition, thrombus formation, calcification, presence/organization of collagen fibers, dehiscence, and graft rejection were examined. Graft assessment was done for the entire circumference of the vessel using a semi-quantitative scale described by Veiga et al. (13), which is based on the severity of histopathologic changes (grade 1: absent, 0-10%; grade 2: mild, 11-25%; grade 3: moderate, 26-50%; grade 4: severe, 51-100%).

### Statistical analyses

Data were analyzed using SPSS v18.0 (IBM, USA) and are reported as the mean±SD. Non-parametric data were analyzed with the Kruskal-Wallis test. Parametric data were assessed by analysis of variance and two-way repeated measures analysis of variance (two-way RM ANOVA). P<0.05 was considered significant.

## Results

**Figure 1:**
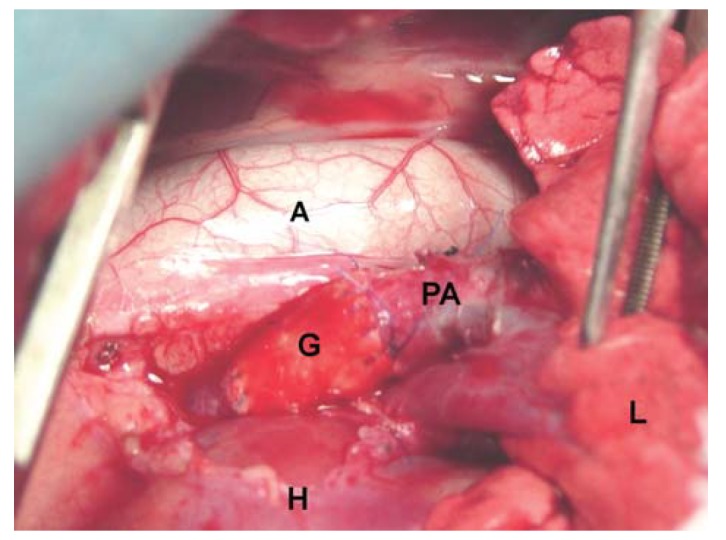
Photograph showing replacement of the left pulmonary artery with a graft. PA: pulmonary artery; A: aorta; G: LyoPA graft; H: heart; L: lung.

In all cases, both grafts showed similar consistency to that of a normal PA and allowed for easy passage of the suture. Grafts recovered their form after reperfusion and did not permit leakage of blood from suture holes (Figure 1). Microbiologic cultures tested negative in all cases after 15 days of aerobic incubation. All dogs survived the surgical procedure. No animals showed significant clinical changes.

### Hemodynamic and blood gas analyses

No hemodynamic or blood-gas changes were observed in either group relative to baseline values or between groups (P>0.05, ANOVA) with respect to heart rate, mean arterial pressure, central venous pressure, systemic vascular resistance, shunt, partial pressure of oxygen, partial pressure of carbon dioxide, or pH. All values were within the normal range.

**Figure 2:**
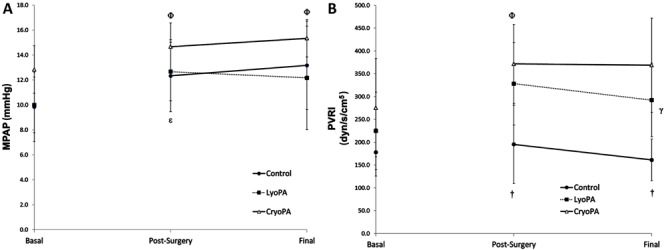
Hemodynamic values for all study groups. Data are reported as means±SD. *A*, Mean pulmonary artery pressure (MPAP; mmHg, left side): Φ: measurements different from basal value in the CryoPA group (P<0.05); ε: measurements different from basal value in the LyoPA group (P=0.021). *B*, Pulmonary vascular resistance index (PVRI; dyn/s/cm^5^), right side): γ: inter-group effects (P=0.004); †: different measurements at the same time between control and CryoPA (P<0.05). Two-way repeated measures ANOVA was used for statistical analyses.

The MPAP in group III increased significantly at the end of the study compared with baseline (P=0.02) and final (P=0.007) two-way RM ANOVA Bonferroni values, but no significant changes were observed upon comparison between groups (P=0.14, two-way RM ANOVA; Table 1, Figure 2).

**Table:**
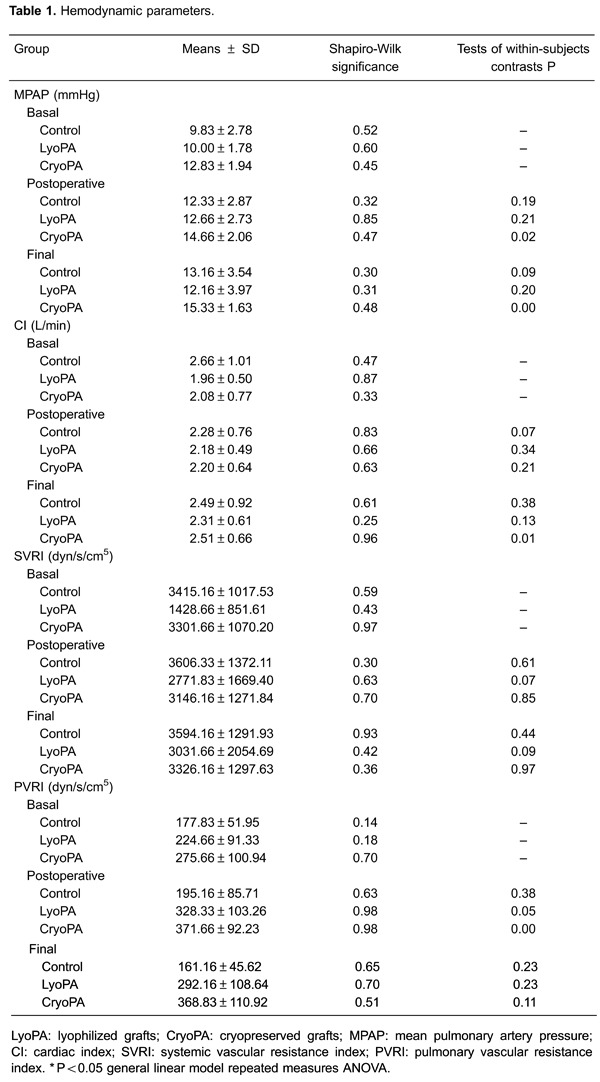

The PVR of groups II and III increased immediately after reperfusion and at study end compared with baseline values. However, the increase shown by group III *versus* group I was significant only when compared after surgery and study end (P=0.016 and P=0.005, respectively, two-way RM ANOVA Bonferroni; Table 1, Figure 2).

### Imaging

**Figure 3:**
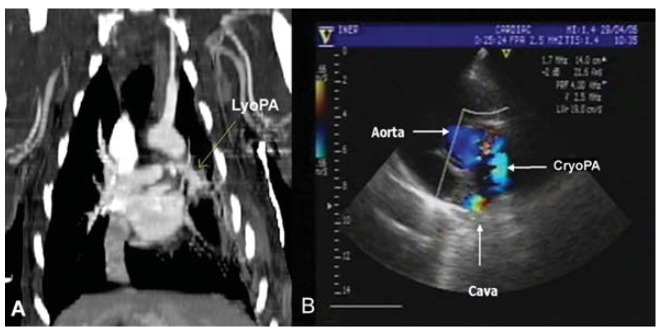
Angiographic and echographic findings at study end. *A*, lyophilized graft (LyoPA); *B*, cryopreserved graft (CryoPA). Patency can be observed in the proximal and distal anastomosis of the pulmonary artery graft.

Radiographic images of all groups did not exhibit any change over the course of the study. Angiography and echocardiography revealed total permeability in 100% of grafts in groups I and II. However, in group III, four dogs (66.6%) had a completely permeable graft (Figure 3), whereas two dogs had a blocked graft.

### Macroscopic evaluation

**Figure 4:**
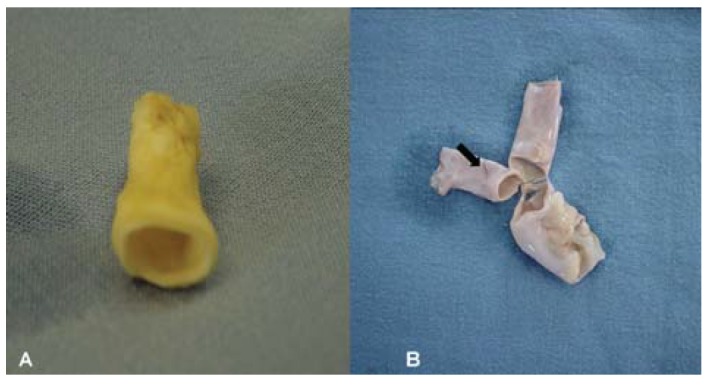
*A*, Pulmonary artery graft after lyophilization. *B*, Pulmonary artery graft showing fractures (arrow) after thawing.

None of the LyoPA grafts and four of the CryoPA grafts had obvious macroscopic changes after rehydration or thawing. Two CryoPA grafts had edge fractures after thawing. At study end, 100% of dogs in group I and II showed complete patency of the artery and graft with good healing of the anastomosis, adventitia and endothelium, and absence of aneurysms, dehiscence or infection. In group III, four dogs (66.6%) had full graft patency with good external and internal healing, and they did not develop aneurysms, dehiscence or infection. However, the remaining two animals (33.3%) had thrombi and fibrous tissue around the graft that obstructed the entire lumen of the graft (Figure 4).

### Microscopic evaluation

**Figure 5:**
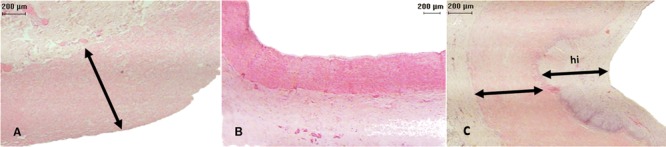
Histologic examination (hematoxylin-eosin staining, ×2.5 magnification) showing: *A*, control pulmonary artery (arrow: artery wall); *B*, a lyophilized (LyoPA) graft, pulmonary artery without alterations; and *C*, a cryopreserved (CryoPA) graft (arrow: artery wall; hi: intimae hyperplasia).

**Figure 6:**
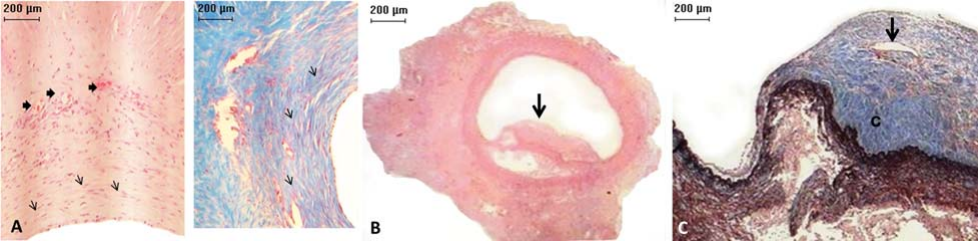
Micrograph of a cryopreserved (CryoPA) graft. *A*, *left*. Pulmonary artery with formation of new vessels (thick arrows) and fibroblast proliferation (thin arrows), hematoxylin-eosin, ×10 magnification. *A*, *right*. Pulmonary artery with an increased number of collagen fibers (arrows). Masson's trichrome staining, ×10 magnification. *B*, Artery with a thrombus (arrow) that partially blocked its lumen. Hematoxylin-eosin, ×2 magnification. *C*, Artery with a thrombus (detail) with area of recanalization (arrow) and collagen deposition (c). Masson's trichrome staining, ×2.5 magnification.

All (100%) LyoPA grafts and four (66.6%) CryoPA grafts, after rehydration and thawing, respectively, showed a normal histologic structure, whereas two cryopreserved grafts exhibited areas with loss of continuity of the endothelium. At study end, the endothelium, intima, and adventitia in all animals of group I were normal, with appropriate healing characterized by moderate development of well-organized, thin collagen fibers (Figure 5). In addition, inflammation, thrombi, intimal proliferation, or calcifications were not observed. In group II, 100% of grafts showed mild inflammation but maintained a normal structure of the endothelium, intima, and adventitia. Both anastomoses healed well, with three (50%) grafts showing mild development and disorganization of collagen fibers; however, these findings were moderate in the three (50%) remaining grafts. Two (33.3%) cases showed intimal proliferation and infiltration of inflammatory lymphocytes that was mild in one case and moderate in another. Thrombi or calcifications were not observed. In all layers of CryoPA grafts, three animals (50%) showed mild inflammation; one (16.6%) had moderate inflammation (fibroblasts were present) and two (33.3%) had severe inflammation (lymphocytes and fibroblasts were present). Healing in four animals (83.3%) showed moderate development and disorganization of collagen fibers in all layers of the vessel, whereas the other animals (33.3%) showed severe fibrosis, with complete disorganization of collagen in all vessel structures (P=0.026, Kruskal-Wallis; Table 2). In addition, four (66.6%) CryoPA grafts showed loss of the endothelium and developed mild intimal hyperplasia at distal anastomoses. The remaining two (33.3%) grafts had severe hyperplasia in both anastomoses. In two (33.3%) cases, thrombi and calcification occurred in the distal portion of the graft (Figure 6; Table 2).

**Table:**
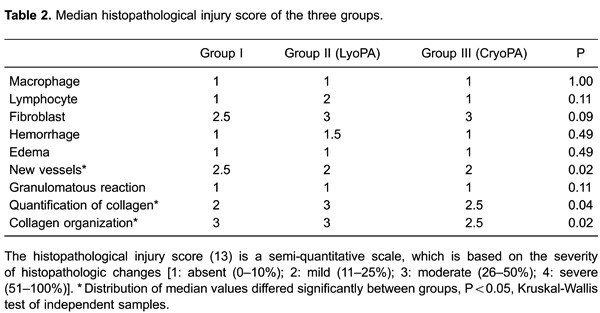

## Discussion

Cryopreserved allografts and lyophilized bovine pericardium grafts treated with glutaraldehyde have been used for heart-valve replacement. They are formed into tubes or patches for replacing intrathoracic vessels because they have good surgical handling. Also, recipients of cryopreserved allografts do not develop a clinically relevant immune response and do not require anticoagulation therapy (6,14). The literature states that glutaraldehyde-pretreated grafts develop calcifications. However, some authors have reported that lyophilization before chemical treatment reduces inflammation, prevents calcification (7), does not alter the mechanical characteristics of the biomaterial, improves its immunogenic properties, and allows graft storage for future use (8).

LyoPA grafts showed similar visual and tactile characteristics to those of a healthy PA after 30 min of rehydration. They showed high hemostatic capacity and excellent hemodynamic response in the immediate postoperative period, and allowed easy passage of the suture because mechanical properties were retained. These findings are consistent with those described by Taniguchi et al. (3), Borgognoni et al. (15), and Flameng et al. (16) with regard to the properties of a lyophilized bovine pericardial bioprostheses. Findings for CryoPA grafts were similar to those described by Gómez-Caro et al. (6), who studied vessel reconstruction after excision of thoracic malignancies using arterial cryopreserved allografts. They reported that these grafts improved the malleability and adaptability of replaced intrathoracic vessels (particularly the PA).

In the present study, microbial cultures of both grafts, after preservation and before use, were negative. An antibiotic was added during the preparation, and both grafts were sterilized for storage with ethylene oxide. Schamún et al. (17) reported that antibiotic addition for procurement of valves, arteries, and pericardium for tissue banks prevents pathogen growth. Furthermore, Leirner et al. (18), and Hafeez et al. (19) studied the prospects for lyophilization of the pericardium, and noted that subjecting lyophilized material to gamma-rays, microwave irradiation, or ultraviolet radiation can eliminate microorganisms (including viruses) if the material is to be used in medical/surgical applications. In the present study, sterilization with a combination of hydrogen peroxide vapor and low-temperature plasma gas prevented the development of microorganisms on LyoPA grafts. This finding is consistent with that described by Olmos et al. (20), who used lyophilized bovine pericardium prostheses for medialization of vocal cords. Conversely, microbiologic cultures of cryopreserved grafts, before they were used as PA grafts, were negative after 15 days because addition of antibiotics and the temperature at which they were maintained (-196°C) prevented the growth of microorganisms in grafts. These findings are consistent with those described by Olmos et al. (21).

The post-perfusion increase in the PVRI and MPAP could have been due to progressive microvascular obstruction caused by post-ischemic lung perfusion. This phenomenon is associated with thrombi formation and vasoconstriction, and impedes blood flow to the vascular bed of the lung, as described by Colombat et al. (22) (who studied the effect of platelet aggregation and oxidative stress on pulmonary post-perfusion hemodynamics). The significant increase in the PVRI and MPAP observed in group III at study end could have occurred because all blood flow went to the pulmonary vascular bed of the right lung due to blocked grafts. These findings are similar to those observed by DellaRocca et al. (23), who evaluated hemodynamic and blood-gas changes during anesthesia in patients with impingement of the left pulmonary artery. This finding has also been described by Bernard et al. (24) while studying pulmonary thromboembolism and endarterectomy.

Different biopreservation methods can affect endothelial cells in different ways. Electron-microscopy studies in lyophilized vessels have shown that endothelial cells and adjacent muscle cells are swollen, with endothelial cells containing many vacuoles and showing moderate damage (25). Conversely, cryopreservation of vascular tissues has been shown to lead to smooth-muscle contractility and to partial loss of the function and tissue of the endothelium.

The most commonly used cryoprotectant for vascular tissues is DMSO (9). In the present study, DMSO and FBS along with slow thawing at 1°C/min were used for cryopreservation assays. Despite this strategy, fractures were observed macroscopically and microscopically in CryoPA grafts that were probably caused by ice formation in the vessel walls during thawing. Also, the mechanical stress that occurs during graft handling may have caused these lesions in all layers of the vessel. These complications have been described clinically and experimentally with use of these grafts (14,26-28). Moreover, there is evidence that these changes are related to freezing injury and are not simply a consequence of exposure to a hypertonic cryomedium (29).

Cryopreservation methods were developed originally for isolated cells. Tissues, however, are multicellular systems containing diverse cell types with differing requirements for optimal preservation. Densely packed cells within a tissue (e.g., arteries) are more likely to be damaged by cryopreservation than cells in loosely packed tissues (e.g., veins). That is, cryopreservation efficacy improves with fewer and less-packed cells (9). In addition to changes in the vascular structure, cryopreservation induces tissue-specific changes in transmembrane signaling, release of intracellular Ca^2+^, sensitivity to Ca^2+^, and Ca^2+^ entry into smooth muscle cells (9,30). These phenomena suggest the need for Ca^2+^-blocking post-implantation therapy or addition of Krebs-Henseleit solution during cryopreservation, which leads to optimal recovery of post-thaw contractile function. However, none of these approaches were used in the present study.

In this work, thrombosis was observed in two CryoPA grafts, but not in LyoPA grafts. In contrast, lyophilization without pretreatment did not elicit significant structural damage to grafts, and grafts maintained their mechanical properties.

Macroscopic and histologic presence of thrombi in two dogs that had CryoPA grafts may have been because the graft structure was damaged, causing an exacerbated inflammatory response, platelet aggregation, and thrombus formation (31,32). Normal histologic characteristics in LyoPA grafts after rehydration revealed that lyophilization, by freezing water and removing ice by sublimation, maintained global morphologic characteristics and protein activity. This finding is consistent with that described by other authors who studied the effect of lyophilization on various tissues used as bioprostheses (3,7,8,15,20,33). Histologic changes observed in group II were probably because lyophilization maintained the matrix structure and graft walls, as several authors who have used lyophilized prostheses (treated and untreated with different chemicals) have reported (3,7,8,16,34). Absence of calcifications in these grafts could have been due to immediate lyophilization after donor procurement with no chemicals added to preserve them. This hypothesis is consistent with results described by Aimoli et al. (7) and Polak et al. (34), who studied lyophilization of bovine pericardium without additional treatment. However, our findings are not in accordance with those described by Taniguchi et al. (3), Flameng et al. (16) and Zuki et al. (8), who suggest that lyophilization reduces inflammation, but not calcification.

In this work, the moderate-to-severe inflammation observed in cryopreserved grafts could have been due to severe fibrosis, with complete disruption of collagen in all vessel structures (as shown in group III), even though normal morphology was maintained after cryopreservation (35). Subsequently, these inflammatory cells may migrate from smooth muscle towards the intima and media. Also, during migration, metalloproteinases degrade collagen and elastin fibers, thereby exacerbating coexisting inflammation and stimulating fibroblasts that survive cryopreservation in the donor and recipient fibroblasts that migrate to the graft, resulting in large quantities of disorganized collagen and intimal hyperplasia (36,37). Our findings are consistent with those described by Komorowska-Timek et al. (36). They studied histologic changes in cryopreserved grafts and found that the main injuries suffered by cryopreserved allografts are persistent infiltration of mononuclear cells (mainly in the adventitia) and intimal thickening by proliferation of myointimal cells the first month after implantation. The inflammatory reaction in the intima, with lymphocyte infiltration and thrombosis, occurs in acute rejection (38-40), as observed in dogs in which the CryoPA grafts were placed.

To summarize, calcification and thrombus formation after graft implantation is influenced by the cryopreservation method and immunogenicity. Further investigations with a longer study time are recommended to ascertain if calcification and thrombi are found in lyophilized grafts.

## Conclusions

Under the experimental conditions described in this work, CryoPA grafts developed potential complications such as thrombi and hemodynamic instability in the pulmonary circulation. LyoPA grafts did not develop thrombi and maintained hemodynamic and blood-gas parameters within normal ranges. LyoPA grafts developed more organized tissue healing (thereby preventing obstruction of vessel lumina), were prepared readily, had adequate strength and flexibility, were easy to manage, and were inexpensive. Our results suggest that LyoPA grafts that are not pretreated with glutaraldehyde could be feasible and are clinically promising.
